# Acute appendicitis and situs viscerum inversus: radiological and surgical approach—a systematic review

**DOI:** 10.1186/s40001-023-01059-w

**Published:** 2023-02-20

**Authors:** Giuseppe Di Buono, Salvatore Buscemi, Massimo Galia, Elisa Maienza, Giuseppe Amato, Giulia Bonventre, Roberta Vella, Marta Saverino, Emanuele Grassedonio, Giorgio Romano, Antonino Agrusa

**Affiliations:** 1 Department of Surgical, Oncological and Oral Sciences, University of Palermo, Via L. Giuffrè, 5, 90127 Palermo, Italy; 2 Department of Radiology, University of Palermo, Palermo, Italy

**Keywords:** Acute appendicitis, Situs viscerum inversus, Midgut malrotation, Kartagener syndrome, Laparoscopic appendectomy

## Abstract

**Introduction:**

Acute appendicitis is one of the most frequent intra-abdominal diseases requiring emergency surgical consult and treatment. The diagnosis of this condition is based on clinical features and radiologic findings. One-third of patients with acute appendicitis present unusual symptoms. There are several circumstances that may cause misdiagnosis and unclear prognostic prediction. Among these, situs viscerum inversus totalis and midgut malrotation can be challenging scenarios, leading to a delay in treatment, especially when these conditions are unknown. We decided to carry on a systematic review of published cases of acute appendicitis in the context of anatomical anomalies.

**Methods:**

We used the MESH terms “appendicitis” AND “situs inversus” AND/OR “gut malrotation” to search for titles and abstracts. Inclusion criteria were patients with clinical and/or radiological diagnosis of acute appendicitis, with conservative or surgical management and with preoperative/intraoperative findings of situs viscerum inversus or gut malrotation. Additionally, previous reviews were examined. Exclusion criteria of the studies were insufficient patient clinical and demographic data.

**Results:**

We included in this review 70 articles concerning 73 cases of acute appendicitis with anatomical anomaly. Patients were aged from 8 to 86 years (median: 27.0 years). 50 were male and 23 were female. 46 patients (63%) had situs viscerum inversus, 24 (33%) had midgut malrotation, 2 (2.7%) had Kartagener’s syndrome, one of them (1.4%) had an undetermined anomaly In 61 patients the anatomical anomaly was unknown previously (83.6%), while 16,4% already were aware of their condition.

**Conclusion:**

Acute appendicitis can occur in association of rare anatomical anomalies and in these cases diagnosis can be challenging. Situs viscerum inversus and midgut malrotation should always be considered in the differential diagnosis of a patient with left lower quadrant pain, especially in younger population. Besides clinical features, it is fundamental to implement the diagnostic progress with radiological examination. Laparoscopic approach is useful to identify and treat acute surgical emergency and it is also a diagnostic tool and can be tailored in order to offer the best exposition of the operatory field for each single case.

## Introduction

Acute appendicitis is one of the most frequent intra-abdominal diseases requiring emergency surgical consult and treatment. The diagnosis of this condition is based on clinical features and radiologic findings. There are many scoring systems that can help to increase the clinical diagnostic accuracy of acute appendicitis [1–3]. Score results should be evaluated in order to guide the decision-making progress toward discharge, observation or surgical management. Sometimes clinical findings are not sufficiently clear and so further investigations like abdominal ultrasound and CT scan are required for differential diagnosis. Approximately one third of patients with acute appendicitis present unusual symptoms such as pain localized outside of the right lower quadrant [4]. There are several circumstances that may cause misdiagnosis and unclear prognostic prediction. Among these, situs viscerum inversus totalis (SIT) and midgut malrotation (MM) can be challenging scenarios, leading to a delay in treatment, especially when these conditions are unknown. Nowadays, even if these anatomical anomalies are rare in the population, there is bigger awareness of their existence. Also, thanks to fetal morphology scan, today it is possible to make an early diagnosis of atypical anatomy [5]. We decided to carry on a systematic review of published cases of acute appendicitis in the context of anatomical anomalies, such as situs viscerum inversus, midgut malrotation and Kartagener’s syndrome. The primary endpoint of this review was to clarify the role of preoperative radiological examination (US and CT scan) for correct diagnosis of acute appendicitis in patients with these anatomical anomalies. The secondary endpoints were to identify the location of pain and surgical management (open versus laparoscopy).

## Methods

**Fig. 1:**
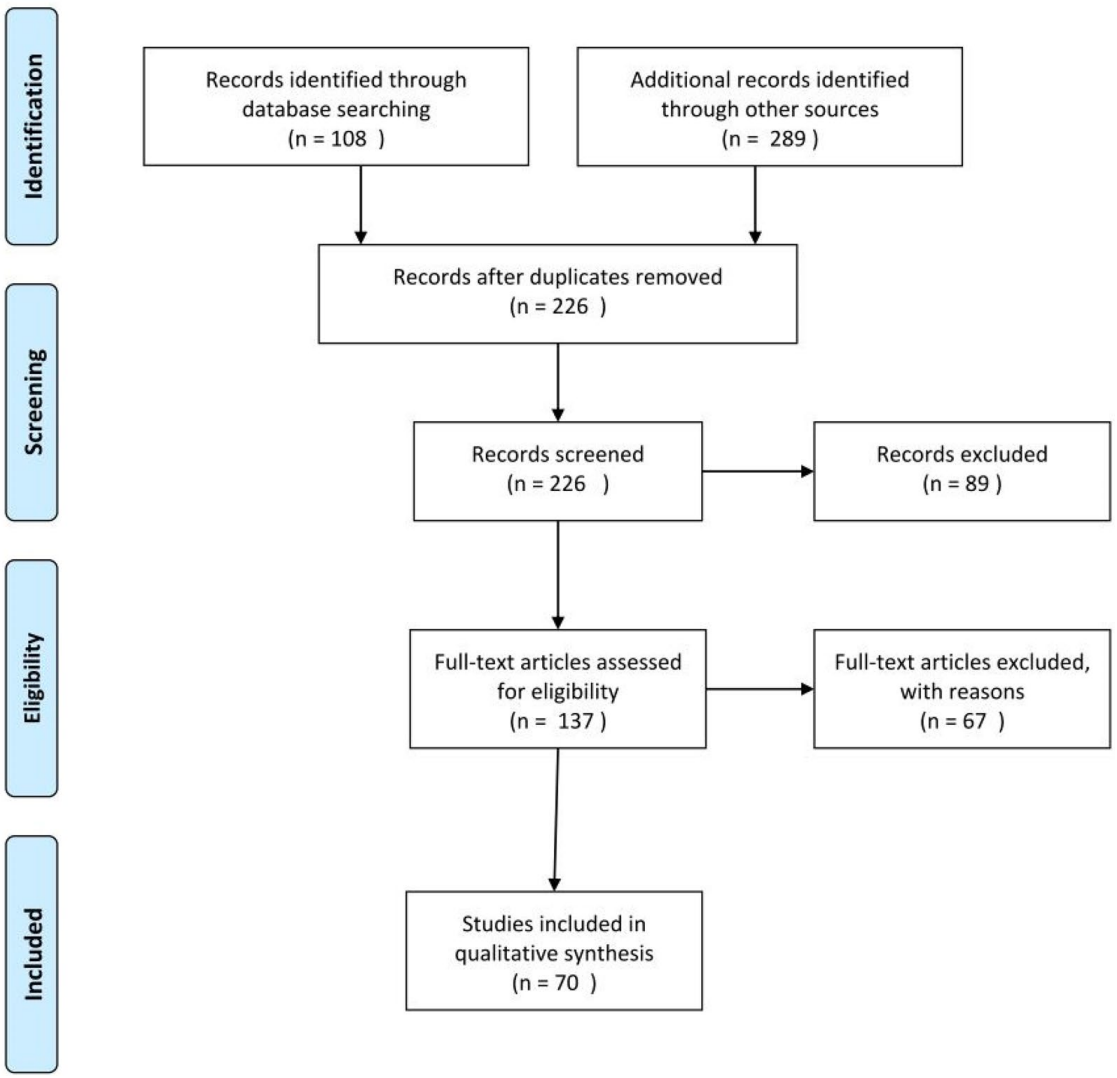
Flow diagram PRISMA for article selection

This systematic review was reported in adherence with the PRISMA statement (Fig. 1) and the study was publicly registered (PROSPERO 2021 CRD42021247073) [6].

### Search strategy and study selection

The search was carried out, independently by two authors, on PubMed, Scopus, ISI Web of Knowledge, Science Direct and Directory of Open Access Journal (DOAJ) databases on April 2021. We considered studies published in English, French and Spanish languages and with available full text. Any discordance was resolved by consensus. We used the MESH terms “appendicitis” AND “situs inversus” AND/OR “gut malrotation” to search for titles and abstracts. Duplicated publications were excluded from the search. At first, titles and abstracts were screened, then the relevant full text articles were retrieved and screened. Inclusion criteria were patients with clinical and/or radiological diagnosis of acute appendicitis, with conservative or surgical management and with preoperative/intraoperative findings of situs viscerum inversus or gut malrotation. Additionally, previous reviews were examined. Exclusion criteria of the studies were insufficient patients clinical and demographic data.

### Data extraction

All data were extracted independently from the full text of articles. We considered the following variables: lead author, year of publication, country, study design, age and sex of the patient, pain location, WBC count, diagnostic radiological tools such as X-ray, abdominal ultrasound or CT scan, time for diagnosis, type of anatomical anomaly, surgical approach. The methodology and context of the included studies were extremely variable, and therefore meta-analysis was not indicated. The analysis of collected data was performed using SPSS software version 13.0.

## Results

Among 226 records, we excluded 89 of them since they were not coherent with our systematic review. Among the remaining 137 articles, we took into consideration those whose full text was available and language was English or French or Spanish, excluding therefore 67 more papers (Fig. 1). We included 70 reports concerning 73 cases of acute appendicitis with anatomical anomaly meeting the above-mentioned criteria. The article types were as follow: 67 case reports, 4 case reports with review of literature, 1 review of literature, 1 observational study, 1 retrospective cohort study. Clinical and pathological characteristics of the 73 patients are summarized in Table 1. Patients were aged from 8 to 86 years (median: 27.0 years). Fifty were male (median: 30.0 years, range: 9–86 years) and 23 were female (median: 24.0 years; range: 8–60 years). 46 patients (63%) had situs viscerum inversus, 24 (33%) had midgut malrotation, 2 (2.7%) had Kartagener’s syndrome, one of them (1.4%) had a left-sided appendicitis (undetermined anomaly) due to a mobile ascending colon and inflammatory appendix adhering to the descending colon over the left lower abdomen. In this condition, the position of the ascending colon mimicking a MM but the intraoperatively exploration excluded this anatomical condition. In 61 patients the anatomical anomaly was unknown previously (83.6%), while the 16,4% of patients already were aware of their condition, either because they found out during previously surgical operations or previous radiological examinations performed for other reasons. We observed that the majority of misdiagnosed cases were higher in the past, while nowadays early diagnosis of these anatomical anomalies is more frequent, presumably thanks to the fetal morphology ultrasound and the larger use of radiological examination in the population. According to location of the symptoms, 69.9% of patients complained left lower quadrant pain, 8.2% presented right lower quadrant pain, 13.7% peri-umbilical pain and 8.2% diffuse abdominal pain. Time of diagnostic of the anatomical anomaly was as follow: 83.6% of the cases were diagnosed preoperatively thanks to clinical suspicion and radiological findings; 16.4% were diagnosed intraoperatively, althought in one case the presence of situs viscerum inversus totalis was confirmed with X-ray of the thorax. The preoperative diagnosis required CT scan in 50.7% of the cases, abdominal ultrasound in 24.7% and X-rays in 13.7%. In 11% of cases, in the past decades, diagnosis was made only based on clinical findings without support of any radiological tool. Open appendectomy was performed in 69.9% of the cases; in one case the patient was 20 weeks pregnant [7]. Laparoscopic appendectomy was performed in 20 patients (27.4%); among these, in two cases appendectomy was combined with cholecystectomy [8, 9]; in one case the extracorporeal appendectomy was performed [10]; in another case single port incision laparoscopic appendectomy was achieved [11]. There was one case of conversion to open surgery due to technical reason [12]. At last, in two cases surgery was not performed, but patients were treated conservatively with antibiotic therapy or radiologically guided drainage of abdominal collection [13, 14].

**Table 1:** The articles selected for this review with clinical and pathological characteristics of the 73 patients

Author	Year	Country	Age	Sex	Pain location	Imaging	Time of discover	Surgery	Type of anomaly	Comments
Courtney AD [43]	1931	UK	21	F	Right	none	Intraop	Open	SIT	
Scopinaro AJ [44]	1932	Spain	30	M	Right	RX	Intraop	Open	SIT	
Mason JT [45]	1933	USA	13	F	Left	none	Preop	Open	SIT	
DePol G [46]	1933	UK	35	M	Right	RX	Preop	Open	SIT	
Minne J [47]	1933	France	12	M	Left	none	Intraop	Open	MM	
Pol ZV [48]	1935	Russia	8	F	Right	RX	Preop	Open	SIT	
Votta EA [49]	1936	Argentina	15	F	Left	RX	Preop	Open	SIT	
Block FB [50]	1937	USA	26	F	Right	none	Intraop	Open	SIT	
Winter B [51]	1953	Canada	46	M	Central	none	Preop	Open	SIT	
Craig RD [52]	1962	UK	47	M	Left	none	Intraop	Open	SIT	
Gibbons J [53]	1962	UK	16	F	central	none	Intraop	Open	SIT	
Pillay SP [54]	1976	South Africa	32	M	central	RX	Intraop	Open	SIT	
Du Toit DF [55]	1986	South Africa	20	M	Left	RX	Preop	Open	SIT	
Garg P [56]	1991	India	50	M	Left	RX	Preop	Open	MM	
Nisolle JF [57]	1995	Belgium	9	M	Left	CT scan	Preop	Open	MM	
Janchar T [14]	2000	USA	36	M	Left	US	Preop	No	SIT	
Djohan RS [8]	2000	USA	20	F	Left	US	Preop	Laparo	SIT	Lap chole
Bider K [58]	2001	Switzerland	27	F	Left	CT scan	Preop	Open	MM	
Franklin ME [9]	2001	Mexico	25	F	Left	US	Preop	Laparo	SIT	Lapa chole
Ratani RS [59]	2001	USA	8	F	Other	CT scan	Preop	Open	MM	
Nelson MJ [60]	2001	USA	42	M	Left	CT scan	Preop	Open	SIT	
Hollander SC [61]	2002	USA	9	M	Left	CT scan	Preop	Open	MM	
Hitoshi F [62]	2005	Japan	13	M	Left	CT scan	Preop	Open	MM	
Hou SK [23]	2005	Taiwan	58	F	Left	CT scan	Preop	Open	MM	Long appendix
Hou SK [23]	2005	Taiwan	48	M	Left	CT scan	Preop	Open	SIT	
Ucar AE [63]	2006	Turkey	22	M	Left	US	Preop	Open	SIT	
Tiwari A [64]	2006	UK	30	F	Other	US	Preop	Open	SIT	
Lee MR [65]	2006	South Korea	43	M	Left	CT scan	Preop	Open	MM	
Golash V [10]	2006	Oman	40	M	Left	CT scan	Preop	Laparo	SIT	Extracorporeal appendectomy
Welte FJ [66]	2007	USA	46	M	Left	CT scan	Preop	Laparo	MM	
Ahmed JU [67]	2007	Bangladesh	50	M	Other	US	Preop	Open	SIT	
Adeniyi AE [18]	2008	Nigeria	32	F	Other	none	Intraop	Open	SIT	
Israelit S [68]	2008	Israel	51	M	Central	CT scan	Preop	Open	MM	
Huang SM [69]	2008	Taiwan	60	F	Central	CT scan	Preop	Open	SIT	
Boyle E [70]	2008	USA	42	M	Central	CT scan	Preop	Laparo	SIT	
Ryen C [13]	2009	USA	23	F	Left	CT scan	Preop	No	MM	Epiploic appendagitis
Akbulut S [4]	2010	Turkey	25	F	Left	US	Preop	Open	SIT	
Elmadi A [71]	2010	Morocco	15	M	Other	US	Preop	Laparo	MM	Common mesentery
Akbulut S [22]	2010	Turkey	16	M	Left	US	Preop	Open	SIT	
Akbulut S [22]	2010	Turkey	17	F	Left	US	Intraop	Open	SIT	
Perera WR [72]	2010	Australia	46	M	Left	CT scan	Preop	Laparo	SIT	
Seifmanesh H [73]	2010	Iran	24	F	Left	ECO	Preop	Open	SIT	
Pillow MT [74]	2010	USA	37	F	Left	CT scan	Preop	Open	SIT	
Bertaud S [75]	2010	UK	30	M	Left	CT scan	Preop	Laparo	Kartagener	
Kashif A [76]	2010	Pakistan	24	F	Left	US	Preop	Open	Kartagener	
Cisse M [77]´	2010	Africa	20	M	Left	RX	Intraop	Open	SIT	
Patel RB [17]	2011	India	28	M	Left	ECO	Preop	Laparo	SIT	
Oh JS [19]	2012	Korea	86	M	Central	CT scan	Preop	Laparo	SIT	
Chih-Ying Y [78]	2012	Taiwan	50	M	Left	CT scan	Introp	Open	ndd	
Moll JL [79]	2013	USA	47	M	Left	CT scan	Preop	Open	MM	
Versluis J [80]	2014	Netherlands	18	F	Left	CT scan	Preop	Laparo	SIT	
Bhagavan Naik M [81]	2015	India	16	M	Left	US	Preop	Laparo	SIT	
Shekhar A [12]	2015	Australia	10	M	Left	CT scan	Preop	Laparo	MM	Conversion to open
Sidibé K [82]	2016	Morocco	31	M	Left	CT scan	Preop	Open	MM	Common mesentery
Üçüncü MZ [7]	2016	Turkey	17	F	Left	US	Preop	Open	SIT	Pregnant 20W
Rajkumar JS [11]	2016	India	22	M	Left	RX	Preop	Laparo	SIT	SILS
Evrimler S [83]	2016	Turkey	29	M	Left	CT scan	Preop	Open	MM	
Evrimler S [83]	2016	Turkey	29	M	Right	CT scan	Preop	Open	MM	
Gulacti U [15]	2017	Turkey	20	M	Left	CT scan	Preop	Open	SIT	
Villabona AN [84]	2018	Columbia	23	M	Central	CT scan	Preop	Open	MM	
Saliba C [21]	2018	Lebanon	27	M	Left	CT scan	Preop	Laparo	MM	
Zengin E [85]	2018	Turkey	13	M	Left	CT scan	Preop	Open	MM	
Kong FB [86]	2018	China	75	M	Left	CT scan	Preop	Open	MM	
Castillo-Gonzàlez A [87]	2018	Mexico	49	M	Left	CT scan	Preop	Laparo	MM	
Shilling Bailey K [88]	2019	USA	40	M	Left	CT scan	Preop	Laparo	SIT	
Yeni M [89]	2019	Turkey	48	F	Left	CT scan	Preop	Open	SIT	
Keli E [90]	2019	Ivory Coast	34	M	Left	CT scan	Preop	Laparo	SIT	
Agrawal V [91]	2020	India	24	M	Other	RX	Intraop	Open	SIT	
Cembraneli PN [92]	2020	Brazil	29	M	Left	CT scan	Preop	Open	SIT	
Di Buono G [93]	2020	Italy	23	M	Left	CT scan	Preop	Laparo	SIT	
Çıkı K [94]	2020	Turkey	15	M	Right	US	Preop	Open	SIT	Torsion of spleen
Kharel H [95]	2020	Nepal	32	M	Left	CT scan	Preop	Open	MM	
Arid K [96]	2020	Egypt	28	M	Central	US	Preop	Laparo	SIT	

## Discussion

Among patients referring to emergency room with abdominal pain, acute appendicitis is still one of the most common conditions requiring emergency surgery with an incidence between 4 and 8% [15]. Diagnosis of acute appendicitis can be supposed considering physical symptoms and clinical history of the patient, experience of the surgeon, laboratory tests and radiological findings. There are many scoring systems that can help to increase the clinical diagnostic accuracy of acute appendicitis, such as Alvarado Score, modified Alvarado Score [1], Ohmann Score [2] and RIPASA [3]. Score systems are useful for stratifying patients with acute abdominal pain and suspected acute appendicitis. Score results should be evaluated in order to guide the decision-making progress toward discharge, observation or surgery. Diagnosis of acute appendicitis is not always straightforward, and mortality and morbidity of this condition may increase when surgical treatment is delayed [16]. Misdiagnosis is more likely to occur when patient present atypical symptoms, such as pain in unexpected location. This circumstance can happen since appendix may assume variable anatomical position: retrocecal, subcecal, preileal, postileal, pelvic, subhepatic, mesoceliac, left-sided, projection of right-sided long appendix into the left lower quadrant area [17]. Differential diagnosis of left lower quadrant tenderness is challenging when left-sided acute appendicitis occurs, and it includes diverticular disease, primary epiploic appendagitis, acute pancreatitis, mesenteric ischemia, but also genitourinary tract disorders like pelvic inflammatory disease (PID), ovarian torsion, ectopic pregnancy, epididymitis, prostatitis, testicular torsion, cystitis [18, 19]. Finally, non-specific abdominal pain (NSAP) is also an occurrence to be considered in differential diagnosis of acute abdominal pain [20]. Left-sided acute appendicitis may occur in association with anatomical anomalies, such as situs viscerum inversus totalis (SIT) and midgut malrotation (MM) or in the context of a syndromic scenario such as Kartagener’s syndrome [21], which can complicate diagnostic process and management of these patients [22]. Situs viscerum inversus totalis (SIT) is a condition characterized by a mirror reversal of the normal asymmetrical arrangement of the viscera and the incidence of this anomaly is approximately of 1/8000–25,000 live births [23–26]. SIT is a rare autosomal recessive or in some cases autosomal dominant congenital disease consisting in developmental defect during embryogenesis. Most of the patients affected by SIT are asymptomatic, with normal life expectancy. SIT can occur in combination with primary ciliary dyskinesia, also known as Kartagener’s syndrome, which involves mutations that disrupt motile cilia [24]. Kartagener’s syndrome is characterized by the following trilogy: dextrocardia, recurrent sinusitis and bronchiectasis; male patients are almost infertile because of immobile spermatozoa. The incidence of this autosomal recessive syndrome is about 1/30,000 live births [27]. Midgut malrotation (MM) consist in a rotation anomaly of the embryonic bowel [28]. There are different types of MM: non-rotation, incomplete rotation, reverse rotation and anomalous fixation of the mesentery [29]. MM is caused by genetic mutation in the gene BCL6 affecting the signaling pathway for intestinal rotation. Thus, it is characterized by a non-rotation of the primitive intestinal loop around superior mesenteric artery axis. Incidence of MM is about 1/6000 live births [28]. The most common type of rotational anomalies is non-rotation. In most of the cases it is a silent anomaly; it can also be associated with other congenital anomalies such as congenital heart disease (like heterotaxy), congenital diaphragmatic hernia, omphalocele, intestinal atresia and complex anorectal malformation [30]. Patients with MM usually have a good prognosis and life expectancy. The incidence of acute appendicitis associated with SIT or MM is rare, approximately between 0.016 and 0.024% [31, 32]. In our review of literature, we could observe a prevalence of this condition in males (68.5% of the examined sample), with a median age for both sexes of 27 years. In the majority of cases the anatomical anomaly was unknown (83,6%) although today it has become more and more frequent to discover anatomical defects beforehand thanks to fetal morphology ultrasound [5]. The primary endpoint of this review was to clarify the role of radiological examination for diagnosis of anatomical condition like SIT and MM in patients with acute appendicitis. We registered that the worldwide diffusion of abdominal US and CT scan dramatically improve the diagnosis and knowledge of these uncommon conditions. So, we can observe that if we considered the reports since 1995 (59 cases of 73, 81%) the diagnosis of anatomical anomalies was preoperatively in 55 cases, respectively, with the use of CT scan (n. 38, 64.4%), abdomen US (n. 17, 29%) and X-ray (n. 1; 1.7%). Only 4 patients had an intraoperative diagnosis of SIT or MM. One of the secondary endpoints was to identify the location of pain because, as above-mentioned, many score systems used for diagnosis of acute appendicitis considered this symptom, In our review the most of the patients referred to emergency department with left lower quadrant pain (69.9%). In the other cases pain was localized in other abdominal areas, causing diagnostic difficulties. Blegen et al. [33] in 1949 reviewed 144 cases of patients with SIT who were submitted to surgical procedures; among these, 77 patients had acute appendicitis and the site of maximum pain was located in left lower quadrant only in 23 cases. This evidence stresses the fact that clinical presentation alone may be misleading and further investigations are mandatory. Besides clinical features, diagnosis of acute appendicitis in patients with SIT or MM may be based on electrocardiogram, which can be particularly useful when a dextrocardia is present, but mostly on abdominal ultrasound and CT scan. As we noted in our review, the X-ray investigation was useful in few cases and in the older decades, while the CT scan was the most accurate tool for correct diagnosis (59% of the cases). In the past X-rays were helpful to detect dextrocardia and right-sided gastric bubble. More recently, ultrasound is widely used when acute appendicitis is suspected, but it has several limitations, such as it is operator-dependent and can be ineffective in patients with high BMI or in case of meteorism. The sensitivity of CT scan in acute appendicitis is 94% [34]. The pathognomonic CT scan signs of acute appendicitis are the following: distended appendix, fluid-filled, measuring more than 6 mm in diameter in right lower quadrant [35]. Ben Ely et al. [29] describe the most frequent findings of intestinal malrotation at CT abdominal scan such as abnormal right-sided position of duodeno-jejunal junction, right-sided location of small bowel and left-sided location of colon with ceacum on the left, abnormal superior mesenteric artery (SMA)/superior mesenteric vein (SVM) relationship with SMV positioned to the left of SMA instead of to the right of the artery, and hypoplasia of the uncinate process of the pancreas. In the case of SIT, a left-sided liver and a right-sided spleen and stomach are fundamental clues for the correct diagnosis. In 15.1% of the cases of this review the diagnosis was intraoperative either because there was not the opportunity to perform a CT abdominal scan or because the CT scan findings were not conclusive. We can retain that the risk of false diagnosis can be reduced with the effective use of CT scan, especially when atypical clinical features are present. The last endpoint of this review was the evaluation of surgical management of these patients. As known laparoscopic appendectomy is the standard therapeutic treatment of acute appendicitis. The advantages of this technique are rapid post-operative recovery, shorter hospital stay, less surgical stress and lower post-operative complications [36]. Furthermore, laparoscopic appendectomy represents a valuable tool when clinical and radiological findings are unclear and the appendix is in a rare anatomical position avoiding large incisions needed for adequate access. Laparoscopy allows the inspection of all abdominal cavity, consenting to confirm the initial diagnostic suspect and to recognize other pathological findings [37, 38]. Standard laparoscopic appendectomy can be modified and tailored for patient with SIT or MM [39]. In our review 20 patients (27.4%) underwent laparoscopic appendectomy and only in one case conversion to open surgery was required. Palanivelu et al. [16] in 2007 reviewed 18 cases of acute appendicitis in patients with appendix in an abnormal position, highlighting about the feasibility and the advantages of laparoscopic approach for these conditions, included SIT. Akbulut et al. [4] in 2010 reviewed 95 cases of left-sided appendicitis, and 8 of them were treated with minimally invasive approach. In these cases the authors described the advantages of laparoscopy in differential diagnosis and surgical treatment, but with several difficulties related to different operating field with “mirror image” and reverse laparoscopic view that can be represent a technical challenge also for experienced surgeon. There is no standard position for trocars insertion in these peculiar cases and the surgeon should modify port placement following the main principles of laparoscopy such as triangulation and ergonomy [40–42].

## Conclusions

Acute appendicitis can occur in association with rare anatomical anomalies and in these cases diagnosis can be challenging. SIT and MM should always be considered in the differential diagnosis of a patient with left lower quadrant pain, especially in younger population. Besides clinical features, it is fundamental to implement the diagnostic process with radiological examination. The diffusion of abdominal US and CT scan significantly increased preoperative diagnosis of acute appendicitis in patients with SIT and MM. Even though abdominal ultrasound is a useful exam when acute appendicitis is suspected, sometimes it is not effective or inconclusive. CT abdominal scan may be a reasonable step to make in order to achieve the correct diagnosis when doubtful clinical and ultrasound findings are present. The role of preoperative imaging is even more important considering that in less than 70% of cases pain is localized to the left lower quadrant of the abdomen. Finally, laparoscopic approach is helpful to identify and treat acute surgical emergency and can be tailored in order to offer the best exposition of the operatory field for each single case. Although laparoscopic treatment of acute appendicitis has been practiced since the 1980s and several studies have clarified the advantages of the laparoscopic approach for this pathology from this literature review, it was found that most of these patients with anatomical abnormalities are still treated with open approach.

## Data Availability

The datasets used and/or analyzed during the current study are available from the corresponding author on reasonable request.

## Abbreviations

SIT: Situs viscerum inversus totalis
MM: Midgut malrotation
NSAP: Non-specific abdominal pain
RIPASA: Raja Isteri Pengiran Anak Saleha Appendicitis score
